# Inverse Design of Nanophotonic Devices Using Generative Adversarial Networks with the Sim-NN Model and Self-Attention Mechanism

**DOI:** 10.3390/mi14030634

**Published:** 2023-03-10

**Authors:** Xiaopeng Xu, Yu Li, Liuge Du, Weiping Huang

**Affiliations:** School of Information Science and Engineering, Shandong University, 72 Binhai Road, Qingdao 266237, China

**Keywords:** inverse design, nanophotonics, neural network, generative adversarial network

## Abstract

The inverse design method based on a generative adversarial network (GAN) combined with a simulation neural network (sim-NN) and the self-attention mechanism is proposed in order to improve the efficiency of GAN for designing nanophotonic devices. The sim-NN can guide the model to produce more accurate device designs via the spectrum comparison, whereas the self-attention mechanism can help to extract detailed features of the spectrum by exploring their global interconnections. The nanopatterned power splitter with a 2 μm × 2 μm interference region is designed as an example to obtain the average high transmission (>94%) and low back-reflection (<0.5%) over the broad wavelength range of 1200~1650 nm. As compared to other models, this method can produce larger proportions of high figure-of-merit devices with various desired power-splitting ratios.

## 1. Introduction

With the improvement of nanofabrication technology [1,2] and the demand for high-performance nanophotonic devices, the footprint of these devices is greatly reduced for high integration density. Nanophotonic devices are widely used in imaging [3], optical computing [4], medical diagnosis [5,6], etc. However, due to the many materials involved and the large number of degrees of freedom for the structural variables, the inverse design of nanophotonic devices with desired optical responses is still challenging.

The traditional inverse design methods, such as topology optimization [7], are based on gradient descent and numerical calculation to iteratively optimize the design to approach the target response. However, this method is not heuristic such that if the target response changes, the optimization process must be restarted from the very beginning. The emergence of data-driven methods [8] based on neural networks (NNs) is now showing promising signs for solving these problems and making major breakthroughs in these tasks [9,10,11]. However, the non-uniqueness of inverse scattering is the root cause that hinders the convergence of inverse NN models [12,13]. Fortunately, the generative NN method can address this issue by mapping the structure and its corresponding response spectrum into the latent space [14,15] such that it can ensure structural diversity during the design. Recently, the GLOnet has been proposed to optimize the silicon meta-gratings [16] by combining the generative NN with the adjoint variable method. The variational autoencoder (VAE) has been used to design the metamaterial patterns [14,15,17]. As another popular generative NN model, GANs [18] have an independent discriminator to identify the authenticity of the generated results. By employing its adversarial relationship with the discriminator, the trained generator can produce images with higher quality [19].

Conditional deep convolutional GANs (cDCGAN), as a variant of GANs, can produce specified objects in more detail with the help of conditional variables and convolution layers. It is used to design free-form nanostructures, such as silver antenna [20], diffractive grating [21], hybrid dielectric [22], and so on, according to customer-defined responses. Subsequently, the improved Wasserstein GAN (as WGAN or WGAN-GP [23,24,25]) was proposed to effectively solve the gradient instability and mode collapse problem [26] by using the Wasserstein distance [27] and gradient penalty (GP) [28] in the loss function. For inverse design applications, WGAN is used to generate various meta-structures according to their multifunctional requirements [29]. Although the preparation of the dataset and the training can be time-consuming, the well-trained GAN-based model can quickly generate multiple device structures that can meet the target response.

In this study, we improve the WGAN model on two levels: First, we add a sim-NN [30] after the generator of the WGAN model, as the WGAN-sim model, to effectively improve the ability of generators to design devices with a more accurate desired spectrum. Then, we use the self-attention (SA) mechanism [31,32,33] to assist WGAN-sim, as the SAGAN-sim model, to capture more detailed features of the structure. To determine the performance of the SAGAN-sim model, we use it to explore the nano-patterned silicon-based multi-mode interference (MMI) power splitter for the desired power-splitting ratios. The optimally designed device has an above 94% total transmission and less than 0.5% reflection over the whole bandwidth of 1200~1650 nm. Compared to the WGAN model, the figure-of-merit (FOM) parameter of the designed devices can be improved by 11.86%, which indicates that the SAGAN-sim model may discover more intrinsic connections between the structural parameters and the spectrum responses during the inverse design. To our best knowledge, this is the first time that an SA mechanism was used in nanophotonic applications.

## 2. Method and Results

**Figure 1:**
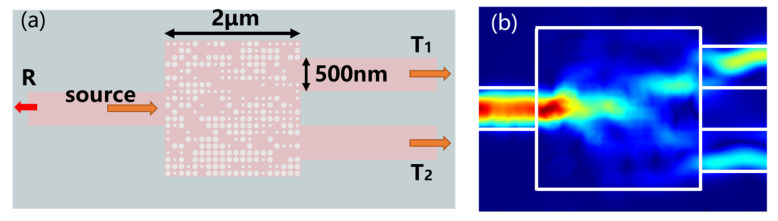
(**a**) The schematic structure of the MMI power splitter, and (**b**) the power distribution in the interference region of MMI at 1550 nm wavelength.

Before describing the method in detail, we first introduce the target device, which is the integrated MMI power splitter with a 2 μm × 2 μm interference region, as shown in Figure 1a. The light enters from the left port and exits from the right ones, which are all 0.5 μm-wide and 2 μm-long straight waveguides connected to the interference region. *T*_1_ and *T*_2_ represent transmissions of the two ports, respectively, and *R* is the reflection back to the input side. For a broad wavelength range (1200~1650 nm), each spectral response has 91 data points in *T* and *R*. The height of the silicon core layer for the splitter is 220 nm and it is covered by the silica cladding on the SOI substrate to be compatible with the conventional CMOS process.

We use the Lumerical FDTD software [34] to model the splitter, whose mesh grid of the interference region is 10 nm, and the mesh type in other regions is non-uniform. The perfect matching layer is used as a boundary of the simulation region, whereas the time step and stability factor are set to 0.0231206 fs and 0.99, respectively. Figure 1b shows the power distribution for the cross-section of the device under TE_0_ mode excitation at wavelength 1550 nm. The interference region is uniformly divided into a 20 × 20 grid matrix, with each grid size of 100 nm × 100 nm. The holes to be etched at any grid points have diameters in the range of 20 to 80 nm, and these diameters are normalized by 80 nm to 0.25~1 to form the 20 × 20 hole matrix (HM). If the normalized diameter value is lower than 0.25 (corresponding 20 nm), no hole will be etched here.

We prepare 10,000 MMI structures with *T*_1_, *T*_2_ and *R* data as the dataset, by using direct binary search (DBS) [35,36] algorithm-guided electromagnetic (EM) simulations. Considering the symmetry of the MMI devices, we flip the structures vertically and switch the transmissions of the two output ports to obtain the final dataset of 20,000 samples. In order to facilitate the DBS algorithm in sweeping the structural parameters, we set the hole diameters as 0 nm, 50 nm or 80 nm to accelerate the data acquisition process. Each sample in the dataset contains a structural configuration (i.e., HM) and the corresponding spectra response *s* (i.e., *T*_1_, *T*_2_ and *R*), which has a total of 273 (=91 × 3) sampling points. Here, 90% of the samples will be used for training and 10% for validation.

### 2.1. WGAN and WGAN-Sim Model

**Figure 2:**
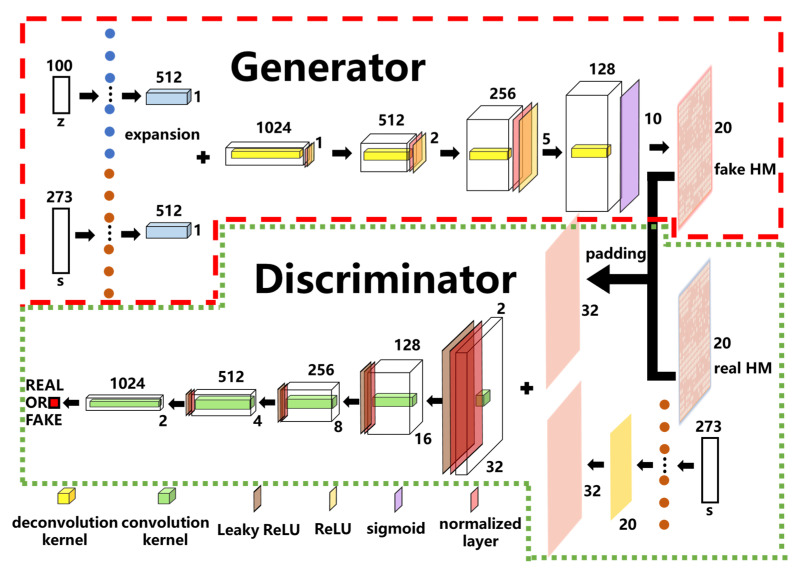
The schematic diagram of WGAN, where s = {*T*_1_, *T*_2_, *R*} is the target response as a conditional vector in the model and *z* is the latent variable. The fake HM is provided by the generator, whereas the real HM is taken from the training set. The dotted layers after *s* and *z* are used for the dimension expansion.

For the WGAN model, we can describe it schematically as in Figure 2, where the generator *G* is enclosed by the red dashed line and the discriminator *D* by the green-dotted one. The numbers at the top and right of the convolution kernels represent the channel and sizes of the output features for each layer, respectively. The target response *s* is used by the generator to produce device structures according to the desired spectrum, whereas the variable *z* is used to construct a latent space so that the structural parameter (i.e., hole matrix, HM) and response *s* can be mapped to it. By altering the values of latent variable *z*, the generator can produce a variety of devices that can have the target response *s*. During the training of *G*, the latent variable *z* with dimensions of 100 × 1 is sampled from the Gaussian distribution and then expanded into a vector of dimensions 512 × 1 × 1 (by the expansion layer as marked by the colored circles). Meanwhile, the target response *s* (consisting of the responses from *T*_1_, *T*_2_ and *R*) of dimensions 273 × 1 is prepared as a conditional vector and is expanded for the next step. Then, *z* and *s* are stacked together to pass a series of deconvolution, normalization and activation layers to obtain the generated (i.e., fake) HM. During training, the fake and real HMs are fed to *D* to discern their differences iteratively, after which, the HM structure and its corresponding target response *s* are padded and stacked for further processing. The convolution, normalization and activation layers are used by *D* to reach a final decision (i.e., fake or real) for each input.

The loss function for *G* is calculated by Equation (1) as minus the expectation value (E) of *D* for all the generated fake HM samples $\tilde{x}$ from the *P_G_* distribution. This is to make *P_G_* as close as possible to the real HM distribution *P_data_*.

(1) $$Loss_{G}=-E_{\tilde{x}\sim P_{G}}\left[D\left(\tilde{x}\right)\right]$$

(2) $$Loss_{D}=Loss_{W}+\lambda Loss_{GP}$$

(3) $$Loss_{W}=-\left(E_{x\sim P_{data}}\left[D\left(x\right)\right]-E_{\tilde{x}\sim P_{G}}\left[D\left(\tilde{x}\right)\right]\right)$$

(4) $$Loss_{GP}=E_{\hat{x}\sim P_{dataG}}\left[{\left({\left\|\nabla_{\hat{x}}D\left(\hat{x}\right)\right\|}_{2}-1\right)}^{2}\right],\;\mathrm{where}\;\hat{x}=\varepsilon x+\left(1-\varepsilon \right)\tilde{x}$$

The loss function for *D* is divided into two parts as given in Equation (2) for the Wasserstein loss ($Loss_{W}$) and the gradient penalty ($Loss_{GP}$), respectively. *λ* is their weighting factor and is set to 10 here to reach the optimum balance. Equation (3) is to calculate the Wasserstein distance between *P_G_* and *P_data_*, such that *D* can be guided by $Loss_{W}$ to distinguish those two. Equation (4) shows the gradient penalty for each sample $\hat{x}$ in order to execute the Lipschitz constraint on *D* [23]. The new distribution *P_dataG_* can be obtained by interpolation between *P_data_* and *P_G_*, with the weighting factor *ε* randomly selected from 0 to 1.

**Figure 3:**
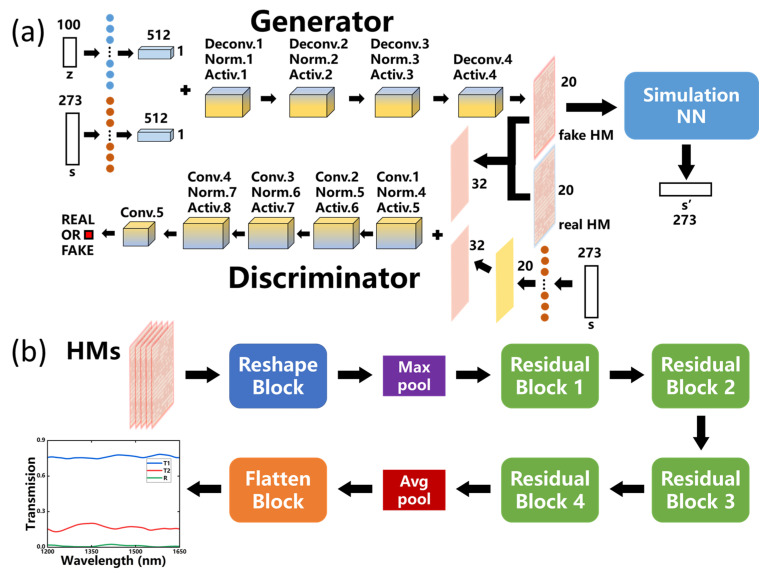
(**a**) The schematic diagram of WGAN-sim, where *s*’ is the response of fake HM predicted by sim-NN. Here, Deconv. means the deconvolution layer, Norm. is the normalization layer, Activ. is the activation function layer, and Conv. is the convolution layer. (**b**) The flowchart of sim-NN.

However, during the training process of WGAN, the mapping of the structure response in latent space may still lack strict restrictions on the response spectra of the generated devices. The discriminator can discern real and fake HMs to train the generator, which can gradually produce more similar structure distributions as compared to the real ones. However, neither the generator nor the discriminator can compare the spectrum responses of the generated devices and the targets, so the generator cannot receive feedback on the spectrum discrepancies during training. In order to avoid this issue, we concatenate a pre-trained simulation NN after *G*, as the WGAN-sim network, to predict the response *s*’ of the fake HM as shown schematically in Figure 3a. The inverse design capability of the generator can be improved by calculating the distance between *s*’ and the target response *s*. Additionally, here, the residual NN based on Resnet-18 [37] is used for the sim-NN, whose flowchart is shown in Figure 3b, as well as the architecture for each building block as listed in Table 1.

During the sim-NN training process, its loss function is defined by the mean squared error (MSE) between the NN-predicted response *s*′ and the EM-simulated one $\bar{s}$, as in Equation (5).

(5) $$Loss_{sim}=MSE\left(\bar{s}-s^{\prime }\right)$$

**Figure 4:**
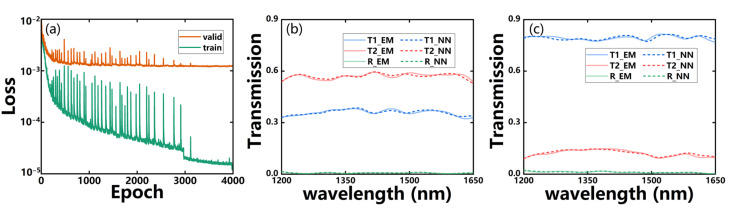
(**a**) The loss evolution during training (green line) and validation (orange line) processes of the sim-NN. (**b**,**c**) Comparisons of the transmission spectra from the EM (solid line) and sim-NN predictions (dashed line) for the two randomly selected MMI splitters from the validation set.

The loss evolution can be shown in Figure 4a for the training and validation. Due to the large quantity and complexity of the dataset, the training is relatively slow, such that the loss curve fluctuates significantly during the first 3000 epochs. However, as the sim-NN prediction accuracy increases, the fluctuation of the validation loss value gradually decreases and eventually disappears after about 3000 epochs. Although the training curve still decreases or even drops suddenly at about 3000 epochs, the validation curve seems flat, which indicates that the model is not improving obviously. Therefore, we stop the training around 4000 epochs as the validation loss stabilizes around 1.2 × 10^−3^. Figure 4b,c shows the responses of two samples randomly selected from the validation set, where the dashed lines are from sim-NN and the solid ones are from EM simulations. The fully trained sim-NN can be seen to predict the spectral response of the device with high accuracy. After this, the sim-NN is fixed for the next step of WGAN-sim training to prevent the deterioration of its prediction accuracy due to influences from *G* and *D*.

(6) $$Loss_{G-sim}=\beta Loss_{G}+Los{s^{\prime }}_{sim},\;\mathrm{where}\;Los{s^{\prime }}_{sim}=MSE\left(s-s^{\prime }\right)$$

The WGAN-sim loss function $Loss_{G-sim}$ for the generator is given by Equation (6), where the first term $Loss_{G}$ is given by Equation (1), and the second one $Los{s^{\prime }}_{sim}$ is given by the MSE of the target response *s* and the sim-NN predicted one *s*′ for the generative device. The discrepancy of these responses can be calculated by $Los{s^{\prime }}_{sim}$, and thus the generator can be trained better. *β* is the weight to balance $Loss_{G}$ and $Los{s^{\prime }}_{sim}$, which is set to 0.01 here for the best training effect. The loss function of *D* involved in the WGAN-sim training process remains the same as in Equation (2).

**Figure 5:**
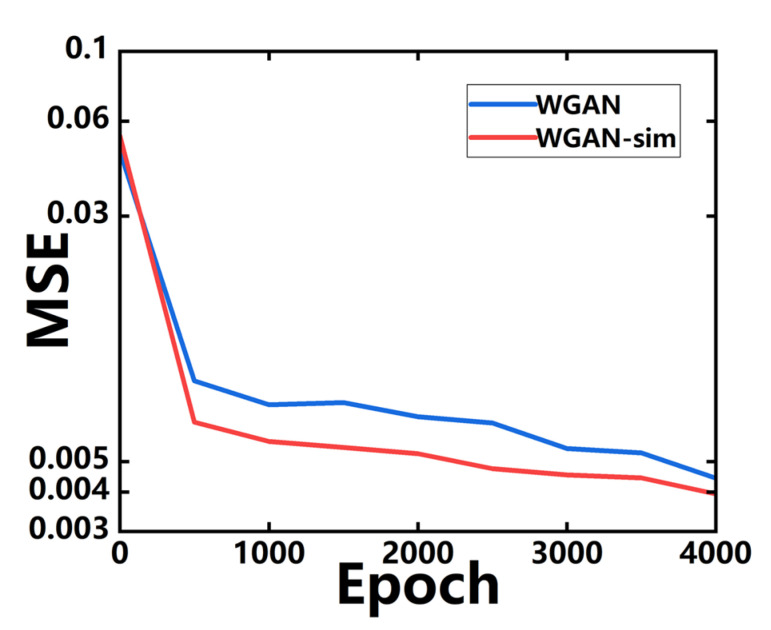
The MSE curves of responses between the target and the generated devices by WGAN (blue line) and WGAN-sim (red line) models. The target responses are from devices in the mini-validation set.

For the simulation efficiency consideration, we randomly select 1000 samples from the validation set, as the mini-validation set, to evaluate the training performance of the above two models. For every 500 training epochs, *s* and *z* are fed into *G* for inverse design, where the generated structures are verified by the EM simulations. As shown in Figure 5, the whole training and validation procedures take 4000 epochs, and the MSE of WGAN-sim drops faster than WGAN.

We further test the performances of the two models to design devices for five different power ratios. For example, in the 5:5 MMI power splitter, we can set the target response to be *T_1_*(*λ*) = *T_2_*(*λ*) = {0.5, 0.5, …, 0.5} and *R*(*λ*) = {0, 0, …, 0}, where the FOM parameter as in Equation (7) is used to indicate the quality of the device response spectrum as compared to the desired one.

(7) $$FOM=1-\left(\sqrt{\frac{1}{n}\sum_{i=1}^{n}{\left(T_{1(i)}^{\prime }-T_{1(i)}\right)}^{2}}+\sqrt{\frac{1}{n}\sum_{i=1}^{n}{\left(T_{2(i)}^{\prime }-T_{2(i)}\right)}^{2}}+\sqrt{\frac{1}{n}\sum_{i=1}^{n}{\left(R_{\left(i\right)}^{\prime }-R_{\left(i\right)}\right)}^{2}}\right)$$

The number of wavelength sampling points *n* is 91 and *i* is its index. *T*_1_’ and *T*_2_’ represent transmissions of the two output ports in the generated devices from EM simulation, and *R*’ is the total back-reflection.

**Figure 6:**
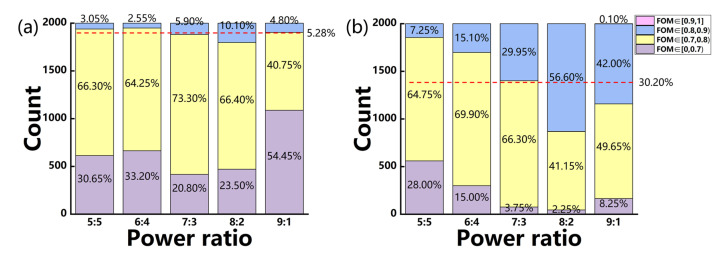
The histogram of FOMs for the generated devices of five different power-splitting ratios, which are generated by (**a**) WGAN, or (**b**) WGAN-sim, respectively. The red dashed line indicates the averaged proportion of the devices with FOM over 0.8 for all five cases.

The comparisons of the WGAN and WGAN-sim models for the inverse design of devices with power-splitting ratios of 5:5, 6:4, 7:3, 8:2, and 9:1 are shown in Figure 6a,b, respectively. The proportion distribution for 2000 generated devices in different FOM ranges is given by each column, whose averaged value for the devices with FOM over 0.8 in all five cases is indicated by the red dashed lines in each figure.

With the help of sim-NN, the performance of the generated device has a significant improvement, where the proportion of devices with an FOM over 0.8 can increase by 24.92%. For the case of the 8:2 power ratio, devices with an FOM over 0.7 can reach 97.75%, which is increased by 21.25% as compared to the WGAN-generated ones.

**Figure 7:**
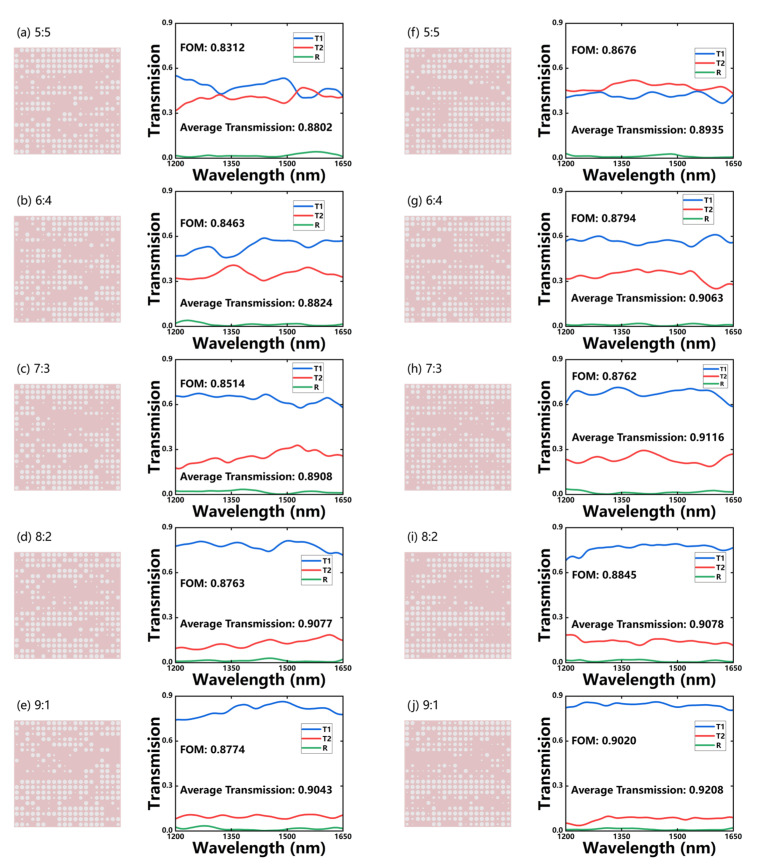
The MMI structures and their transmission spectra for the optimal devices generated by WGAN (**a**–**e**) or WGAN-sim (**f**–**j**), respectively, for five different power-splitting ratios.

Among the 2000 samples generated by WGAN and WGAN-sim for each of the five different ratios, we select the best configuration in terms of FOM as in Figure 7. Both models can generate desirable devices according to the target, whereas the WGAN-sim-designed ones give higher and more stable responses than WGAN (whose average standard deviations are 0.0224 and 0.0302, respectively). The ~10% of energy is scattered and absorbed by the MMI structures during the transmission. The optimal devices from the WGAN-sim model all have an FOM higher than 0.86, and an average transmission over 90%, whereas the back-reflection is less than 1.2% over the whole band. Therefore, sim-NN can effectively boost the performance of WGAN to obtain better inverse-designed MMI structures.

### 2.2. SAGAN-Sim Model

In the above models, high-level features [38], such as the main outline of the interference region, may dominate the receptive fields [39] as the number of convolution and deconvolution layers increases, and then some details of the local information may be blurred. At the same time, due to the limitation of convolution and deconvolution kernel size, it is inevitable that the ability to extract features from the distant pixels of these models is influenced by the (de)convolution layers. During the inverse design process of nanophotonic devices, the local permittivity perturbation [40,41,42] may cause an impact on the overall spectral response, and the interaction for each part of the structures should be fully considered. Therefore, we need the model to be able to learn features more comprehensively by taking into account the relationships between each pixel of the device. During our investigation, we find that the SA mechanism [43,44,45,46,47] can help us solve the above problems properly by capturing the correlation among features in the dataset, even if they are separated by a long distance. These features can be connected between the input and output by proper weights quantitatively with fewer computational resources [43] as compared to other NN layers such as the convolutional and recurrent ones. In the GAN model, the SA mechanism can be used in conjunction with the deconvolution layers to help the generator to produce objects with more detailed information [33]. Furthermore, it also makes the discriminator more effective in detecting the global features from the training data.

**Figure 8:**
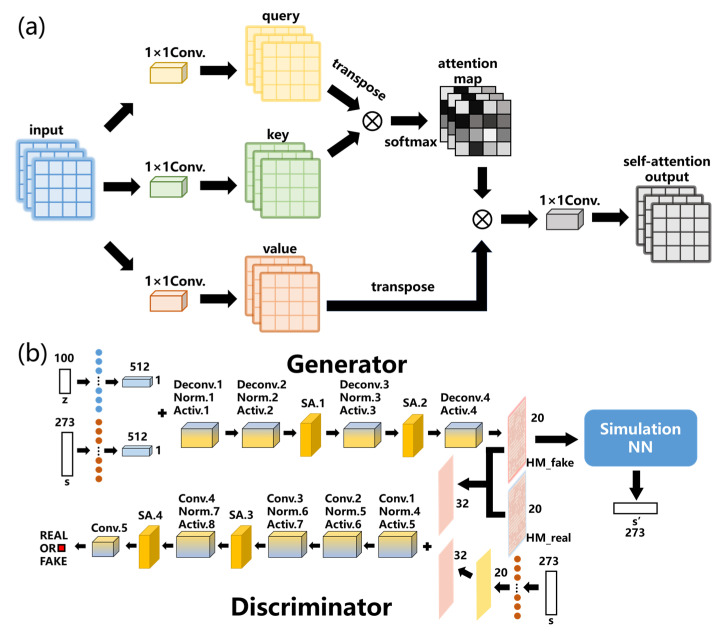
The self-attention mechanism used in SAGAN. (**a**) The schematic diagram of the self-attention layer, where ⊗ represents the matrix multiplication, and (**b**) the framework of SAGAN-sim model.

In order to further improve the ability of the model in focusing more precisely on the local features, we introduce the self-attention layers into WGAN-sim, as the SAGAN-sim model, to generate better devices. The schematic structure of the SA layer is shown in Figure 8a, where features from the previous layers are first processed by the three 1×1 convolution layers to obtain the query, key, and value matrices [43,48]. Then, we calculate the product of the transposed query with the key to be passed through a softmax layer to obtain the attention map [49], as well as a score [50,51] between each and every two features. Finally, the attention map and the value matrix are multiplied and processed by the last convolution layer to obtain the output from the SA layer.

As shown in Figure 8b, for example, the SA layers are added behind the second and third activation function layers in *G*, as well as the seventh and eighth ones in *D*, respectively, such that features are more likely to be extracted with high fidelity as the number of (de)convolution layers increases [52].

**Figure 9:**
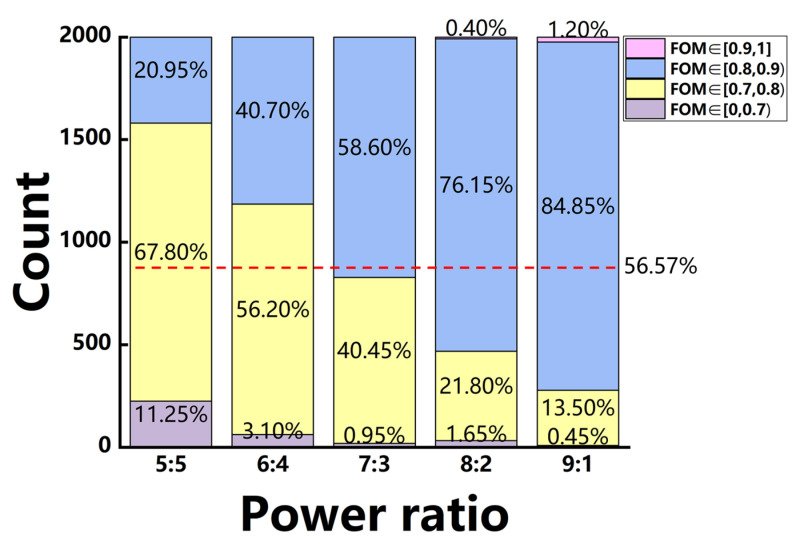
The histogram of FOMs for devices of five power-splitting ratios generated by the SAGAN-sim model. The red dashed line is for the averaged proportion of the devices with FOM over 0.8 for all five cases.

To test the effectiveness of the model, the trained SAGAN-sim is used to generate 2000 devices for each of the desired power-splitting ratios. The proportion statistics of FOM for the SAGAN-sim generated devices can be seen to further improve as shown in Figure 9. The average FOM of all generated devices is more than 0.81, which is about 11.86% higher than that of the WGAN (whose average FOM is 0.72). The proportion for devices with an FOM higher than 0.8, as indicated by the red dashed line, can reach 56.57%, which is an increase of 26.37% and 51.29% as compared to the WGAN-sim and WGAN models, respectively. This large proportion of devices with high FOMs indicates that this model may be able to find more underlying physical characteristics between the structure and spectrum response of the device.

**Figure 10:**
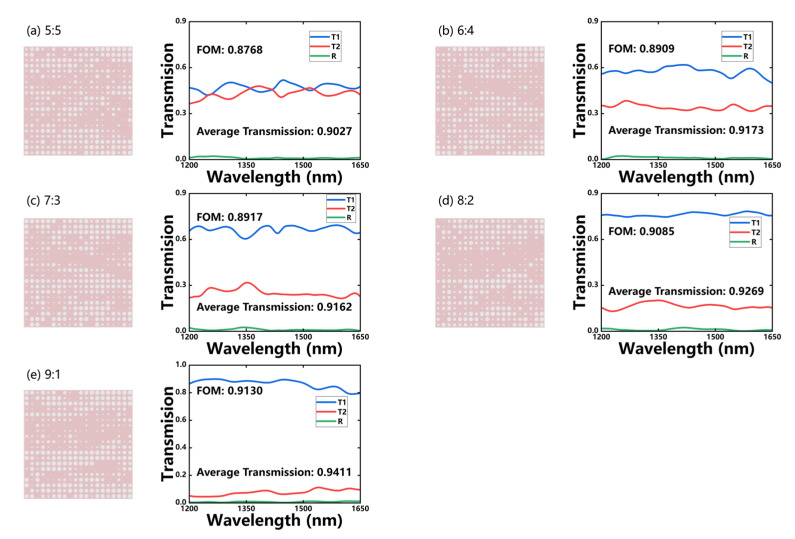
The hole distribution of the designed MMI and their transmission spectra for (**a**) 5:5, (**b**) 6:4, (**c**) 7:3, (**d**) 8:2, and (**e**) 9:1 target splitting ratios, from the SAGAN-sim model.

Here, the SAGAN-sim-designed optimal MMI devices and their spectrum responses for the five ratios are shown in Figure 10. The solid lines are the transmission and reflection spectra, and all of these devices have better FOMs than the previous models. The total transmission of the optimal device is higher than 94%, and the back-reflection can be less than 0.5%. The proportion of absorbed and scattered energy of these devices is about 5~10%.

The programming language and deep learning framework we used to build the NN model are Python 3.7.1 and PyTorch 1.11.0, respectively. For all the models as mentioned in the paper, NVIDIA GeForce RTX 3090 GPUs are used for each GAN and sim-NN training, which take around 20 and 8.5 h, respectively, and the addition of the SA layers will not significantly affect the training time. During the training process, we manually tuned the hyperparameters of these models to ensure the optimized network structure and learning rate, etc. It takes only 6 to 9 s to generate 2000 different high-FOM devices with one single running of the generator. Since the generation process is parallelly conducted for the model, the number of devices generated per inverse design can be set according to the actual demand by controlling the number of target responses and latent variables. The EM simulation of the device is carried out by Lumerical FDTD software and is about 10 s for each structure. We can also use the trained sim-NN instead of the EM simulation software to accelerate the verification process for the generated devices at equivalent accuracy.

## 3. Conclusions

In order to improve the inverse design method based on the generative neural network, the WGAN-sim and SAGAN-sim models are proposed to design nanopatterned MMI power splitters in the photonic integrated circuit. By exploring the global structural parameters in more detail, the SAGAN-sim model can enjoy high accuracy from the self-attention mechanism and the sim-NN to improve the FOM of the generated devices. Compared to the WGAN model, the average FOM for the SAGAN-sim-generated devices increases by 11.86%, whereas the proportion of devices with an FOM over 0.8 is improved by 51.29%. Across the wavelength range from 1200 to 1650 nm, the total transmission of the optimal devices can be over 94% and the reflection below 0.5%. As far as we know, this is the first time that the self-attention mechanism has been used for the inverse design of nanophotonic devices.

Here, we only consider the structural parameters of devices in the two-dimensional cross-section, but this model can be readily applied to more complex nanophotonic devices with more parameters in higher dimensions. In addition to the transmittance and reflectance, the target response can also be the phase spectrum, electric/magnetic field distribution, etc. The NN-based method can also help us reduce the dependency on prior knowledge of the target device. Moreover, the GAN model can generate device structures according to the responses, even if the target response has not appeared in the training process, which indicates that the model can provide intrinsic connections between the device structures and corresponding responses. The method can also be extended to material science, biology, chemistry and other research fields to single out the optimal design according to their desired target properties. The focus of our future study will be on the NN algorithm to train the model with smaller datasets but better accuracy.

## Figures and Tables

**Table 1:** The detailed architecture of the sim-NN.

Block	Input Size	Output Size	NN Parameter
Reshape Block	20 × 20	64 × 32 × 32	Linear ^3^: 100, 1024; Conv ^4^: kernel 7 × 7, channel 64, stride 2
Maxpool ^1^	64 × 32 × 32	64 × 16 × 16	Kernel 3 × 3, stride 2
Residual Block 1	64 × 16 × 16	64 × 16 × 16	[(3 × 3, 64) × 2] × 2, stride: 1, 1, 1, 1
Residual Block 2	64 × 16 × 16	128 × 8 × 8	[(3 × 3, 128) × 2] × 2, stride: 2, 1, 1, 1
Residual Block 3	128 × 8 × 8	256 × 4 × 4	[(3 × 3, 256) × 2] × 2, stride: 2, 1, 1, 1
Residual Block 4	256 × 4 × 4	512 × 2 × 2	[(3 × 3, 512) × 2] × 2, stride: 2, 1, 1, 1
Avgpool ^2^	512 × 2 × 2	512 × 1 × 1	Output size (1, 1)
Flatten Block	512 × 1 × 1	273 × 1	Linear: 512, 4096; Linear: 4096, 273

^1^ Maxpool: max pool layer; ^2^ Avgpool: adaptive average pool layer; ^3^ Linear: fully connected layer; ^4^ Conv: convolution layer.

## Data Availability

Not applicable.
